# The Effect of Superoxide Dismutase Mimetic Drug in an Infertile Patient With Thin Endometrium

**DOI:** 10.7759/cureus.53077

**Published:** 2024-01-27

**Authors:** Sanket Mahajan, Akash More, Shilpa Dutta, Namrata Chaudhary, Neha Nawale

**Affiliations:** 1 Clinical Embryology, Datta Meghe Institute of Higher Education and Research, Wardha, IND; 2 Clinical Embryology, Datta Meghe Institute of Higher Education And Research, Wardha, IND

**Keywords:** reactive oxygen species (ros), autologous platelet-rich plasma (prp), thin endometrium, recurrent implantation failure, infertility

## Abstract

It has been observed that nowadays, millions of couples struggle with infertility, which may be attributed to various conditions. In this case study, a middle-aged couple with a history of recurrent implantation failure (RIF) visited an infertility clinic situated in a rural region in Wardha to seek treatment. The male was normozoospermic. After hysteroscopy, it was noticed that an aggregated level of reactive oxygen species (ROS) was a causative factor for thin endometrium contributing to infertility. The patient was advised to autologous platelet-rich plasma (PRP) treatment and temporary medication. A significant level of amelioration in endometrial thickness was observed, which significantly contributed to the chances of implantation. This resulted in a positive clinical pregnancy outcome for the patient. This case report highlights the fact that a combination of tempol with autologous PRP may contribute to an improved factor for the enhancement of endometrial hyperplasia, which may contribute to an improved in vitro fertilization (IVF) pregnancy outcome.

## Introduction

Throughout the world, infertility affects 15% of couples [1]. While in-vitro fertilization (IVF) and other assisted reproductive technologies (ART) can help with this issue in certain situations, reactive oxygen species (ROS) are known to play a role. The causes of infertility can affect either males or females, or it can affect both. Disorders that interfere with the normal function of the reproductive organs (extragenital etiology), innate or acquired conditions influencing genital etiology, or psychological variables can all impede female reproductive function [2].

In the past, senescence was described as a condition in which cells undergo a practically irreversible proliferative arrest but remain metabolically active [3]. The ability of embryos for implantation requires adequate decidualization. It has recently come to light that senescent endometrial stromal cells have reduced decidualization potential and induce "bystander" quenching of the decidual response in neighboring cells, contributing to dysfunction in the stromal compartment [4]. Previous research by Deryabin et al. has demonstrated that senescent endometrial stromal cells (EnSC) adversely impact the adjacent healthy cells' ability to differentiate into distinct tissues or decidualize by a paracrine effect [3].

The process by which the embryo follows in the footsteps of the uterine endometrial surface, infiltrates the epithelium, and eventually passes through the maternal circulation in order to transform into the placenta is collectively referred to as implantation [5]. However, both the embryo and the endometrium should start a complex process that is specific to a certain time and place before implantation begins. Crosstalk between a competent blastocyst and a receptive uterus can only occur during a specific window of time, known as the "window of implantation" [5].

When injectable progesterone (100 mg/ml for six days) begins to be administered in frozen-thawed embryo transfer (FET) cycles, on the precise day of ovulation, or while human chorionic gonadotrophin (hCG) injections have been administered in newly initiated IVF cycles, the endometrium is considered to be thin if its overall thickness is less than 7 mm [6]. Platelet-rich plasma (PRP) is an autologous platelet plasma concentrate obtained by centrifuging red blood cells from a patient's fresh whole blood. It has anti-inflammatory and regenerative properties [7].

Using PRP is a low-cost, non-invasive method. It is made up of plasma from peripherally drawn blood that has a high platelet concentration [1]. Plasma contains proteins, hormones, and cytokines and stimulates the growth, division, and proliferation of cells [8]. This case report is based on the effect of autologous PRP along with tempol for having a positive clinical pregnancy outcome.

## Case presentation

Patient information

This case study is about a patient who enrolled at an infertility clinic in the state of Maharashtra, India. The individuals who sought infertility treatment were a 36-year-old female and a 40-year-old male patient suffering from primary infertility. The patient was employed as a staff nurse in our hospital. The couple did not exhibit any addictions, such as smoking, tobacco use, or alcohol consumption.

Medical/surgical history

The patient had recurrent implantation failure (RIF) and a history of three intra-uterine insemination (IUI) and two intracytoplasmic sperm injection (ICSI) failed cycles. Asthma, heart conditions, tuberculosis, or hypertension were absent in either partner's medical history. The couple had no prior history of mental or psychiatric illness, and the family history was negative. They sought IVF treatment at our medical facility for the first time.

Physical examination and investigation

The body mass index (BMI) for the female was 24.5 kg/m^2^, and for the male, it was 25.6 kg/m^2^.

Both individuals underwent comprehensive infertility evaluations to determine the underlying cause of their infertility.

According to the husband's semen analysis, the sperm count was reported to be 40 million/ml, semen morphology was 94%, and motility was 64%. The normal morphology of semen is 6%. According to his report, his semen profile indicated normozoospermia.

After an endometrium biopsy, the report revealed high ROS levels, known for causing thin endometrium (<7mm). Her hormonal levels showed abnormalities: the anti-Müllerian hormone (AMH) level was 1 ng/ml, and the follicle-stimulating hormone (FSH) level was normal.

Diagnosis and treatment

This was a case of primary infertility, where the cause was an increased level of ROS leading to a thin endometrium despite normal spermatic parameters in the patient's husband.

The patient underwent a recommended hysteroscopy, and the report indicated a thin endometrium (<7 mm) at the time of implantation. Consequently, a thin endometrium was presumed to be noticeable in the patient with RIF. Treatment alongside autologous platelet-rich plasma (PRP) has been advocated. PRP is well-known for its high platelet concentration, as platelets contain growth factors that promote tissue regeneration and repair. The patient was advised for intracytoplasmic sperm injection (ICSI) and initiated the treatment. Gonadotropin-releasing hormone antagonists (GnRH) and gonadotropin-releasing hormone agonists (GnRH) were used to control ovulation timing and stimulate the development of multiple follicles in the ovary. A trigger was administered for ovum pick-up (OPU). After 36 hours of the trigger, ovarian aspiration was scheduled. Three oocytes were retrieved during OPU (1 MII, 1 MI, 1 GV). ICSI was performed on the same day, resulting in the formation of good-quality cleavage-stage embryos. However, by the third day, the embryos had degenerated.

**Figure 1:**
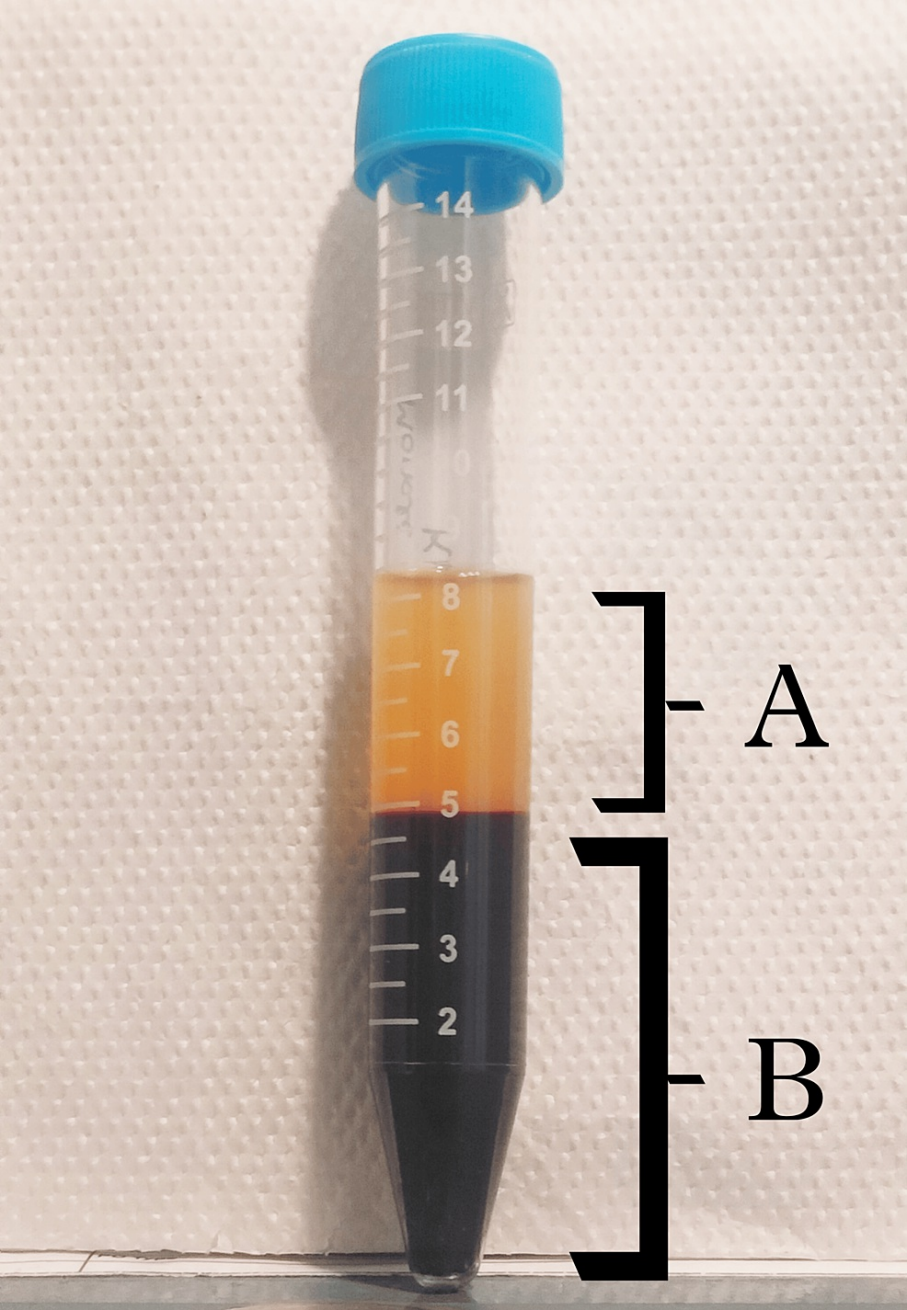
Autologous platelet-rich plasma (PRP) used for the patient A: platelet-rich plasma (PRP); B: remnants of blood cells after centrifugation

**Figure 2:**
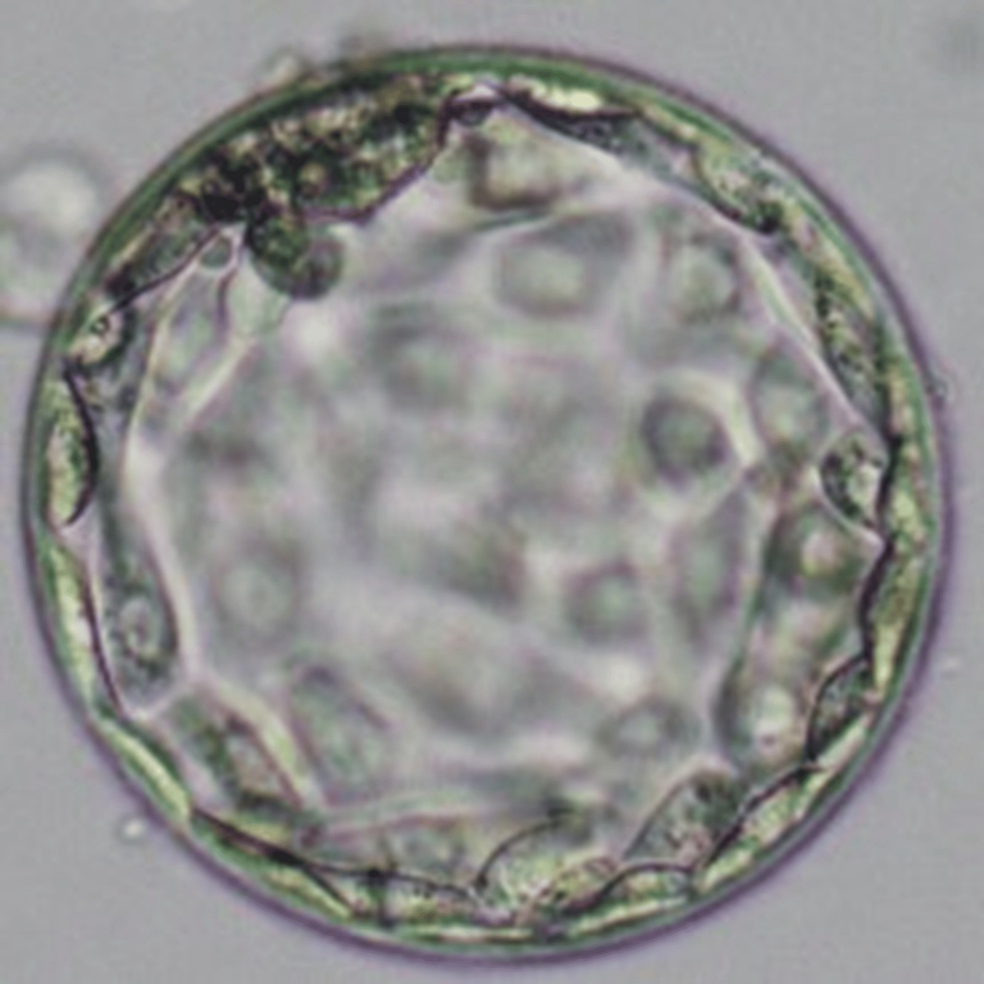
Blastocyst (4AA) transferred to the patient

For the second cycle, the patient was recommended an endometrial biopsy, which revealed a high level of ROS, a notable cause of thin endometrium. The patient was subsequently advised to undergo a three-month treatment with tempol medicine via the intra-vaginal route. After three months, the female underwent another endometrial biopsy, which showed a significant decrease in ROS levels. Two months post-endometrial biopsy, the patient was rescheduled for ICSI along with the utilization of autologous PRP. Follicular fluid screening yielded five oocytes (3 MII, 1 MI, 1 GV). On the same day, ICSI was performed, and autologous PRP was administered two days before embryo transfer (ET). A significant improvement in the endometrial layer was observed, with an increase in endometrial thickness. Good-quality blastocysts (4AA) were transferred during embryo transfer, and the procedure was well-tolerated by the patient. Figure 1 shows the image of PRP used for the patient. Figure 2 denotes the blastocyst (4AA) that was transferred to the patient.

Follow-up

The patient was advised to take prescription medications after the embryo transfer procedure, such as 200 mcg of orally administered progesterone for the next 14 days, to support the growth of the uterine lining and improve implantation. Periodic follow-up visits allowed close observation of the patient as her progress was evaluated. Additionally, the patient received advice on lifestyle adjustments, including suggestions for a nutritious diet, regular exercise, and avoiding possible hazards. Pervasive encouragement and counsel were provided to address any issues or questions that arose throughout the follow-up phase. Paying close attention to the patient's overall condition and the pregnancy's advancement exacerbated their probability of a favorable outcome. Two weeks later, at prepared follow-up appointments, the patient's pregnancy was confirmed by a positive beta-human chorionic gonadotropin (β-hCG) test. The value of β-hCG was reported as 1240 mIU/ml.

## Discussion

The human body utilizes a variety of endogenous antioxidants, including glutathione, ϼ-arginine, coenzyme Q10, lipoic acid, and catalase (CAT), as well as nonenzymatic antioxidants like superoxide dismutase (SOD), CAT, and glutathione peroxidase (GPx), to combat the effects of free radicals and oxidative stress [9]. However, we treated the patient with tempol medicine, which may decrease their reactive oxygen species. The other nonenzymatic antioxidants such as resveratrol, L-ascorbic acid, L-carnitine, N-acetyl-cysteine, cysteamine, quercetin, nobiletin, lycopene, acetonide, mogroside V, phycocyanin and laminarin and new treatment methods. The mentioned antioxidants exhibited good free radical scavenging activity against β-carotene and 2,2-diphenyl1-picrylhydrazyl (DPPH)14 and presented high inhibition in total equivalent antioxidant capacity [10].

The therapeutic properties of autologous PRP for endometrial development in women with thin endometrial tissue had originally been documented by Chang et al. [11]. PRP infusions were administered to five women in that trial who were diagnosed with unsustainable endometrium and had failed to respond well to conventional therapy during the FET cycle. Four of the women reported normal pregnancies, and all of them reported appropriate responses to treatment [11]. This case report focuses on a uniparous geriatric woman.

Infertility followed by autologous platelet-rich plasma (PRP) has recently been at the forefront of extensive study. It has been demonstrated that PRP has an impact on angiogenesis, cell migration, differentiation, proliferation, and tissue regeneration [11,12]. These processes are facilitated by the various cytokines and growth factors that PRP produces when activated. Transforming growth factor-beta, fibroblast growth factor, growth factors resembling insulin 1 and 2, the growth factor for vascular endothelial cells, and epidermal growth factor are examples of certain growth factors and cytokines [12].

The application of senolytic therapy to enhance endometrial receptivity can be challenging. As mentioned earlier, cellular senescence plays a crucial role in the early stages of decidualization by triggering the pro-inflammatory response required to establish a favorable microenvironment and extend the window of time for successful implantation [12,13]. The aging immune system's inability to eliminate senescent embryonic stem cells (ESCs) could disrupt the inflammation crucial for the decidualization process [13].

Cellular senescence in the perivascular niches of the thin endometrium provided evidence that insufficient cell sources caused the endometrium to expand inadequately. Additionally, an indicator of microvascular aging marked by an excess of extracellular matrix (ECM) around the vasculature is the thickening of the wall of the basement membrane [14]. This case report serves as a testament to the fact that the application of PRP along with tempol medication may serve as a revolutionary procedure for the improvement of thin endometrium, thus increasing the chance of achieving clinical pregnancy via ART.

## Conclusions

This case study serves as evidence for patients suffering from senescent endometrial conditions resulting in infertility. The application of tempol medication along with autologous platelet-rich plasma (PRP) improved the elevated reactive oxygen species levels in the endometrium, resulting in successful implantation, validated by a positive beta-human chorionic gonadotropin (β-hCG) test. However, as this article includes only one patient, we recommend further studies with a larger sample size to validate the results of this study.
